# A Rare Case of Anomalous Origin of Left Anterior Descending Artery From Right Coronary Ostium

**DOI:** 10.7759/cureus.18966

**Published:** 2021-10-22

**Authors:** Suganya Karikalan, Munish Sharma, Megha K Chandna, Harish Chandna, Salim Surani

**Affiliations:** 1 Medicine, Karpaga Vinayaga Institute of Medical Sciences, Maduranthagam, IND; 2 Pulmonary Medicine, Pulmonary Asociates, Corpus Christi, USA; 3 Medicine, Texas A&M, College Station, USA; 4 Cardiology, Cuero Regional Hospital, Cuero, USA; 5 Anesthesiology, Mayo Clinic, Rochester, USA; 6 Medicine, Texas A&M University, College Station, USA; 7 Medicine, University of North Texas, Dallas, USA; 8 Internal Medicine, Pulmonary Associates, Corpus Christi, USA; 9 Clinical Medicine, University of Houston, Houston, USA

**Keywords:** right coronary artery (rca), coronary artery angiogram, myocardial infarction type 1, anomalous origin of left anterior descending artery, coronary artery bypass grafting(cabg)

## Abstract

Coronary artery anomalies are rare congenital malformations, most often undiagnosed until late adolescence or adulthood when an angiogram is done for conditions such as myocardial infarction, arrhythmias, heart failure, and sudden cardiac death. Sometimes, an anomalous left coronary artery originating from the right coronary ostium might traverse between the aorta and pulmonary artery and cause chest pain, syncope, myocardial infarction, or sudden death even in younger patients. Here we present a case of an elderly female presenting with chest discomfort on exertion. The coronary angiogram revealed severe triple vessel disease and an ectopic left anterior descending artery arising from the right coronary ostium. After careful evaluation, it was determined that her symptoms were solely due to severe multivessel coronary artery disease (CAD). Thus, she underwent coronary artery bypass surgery for her CAD. It is important to consider anomalous coronary artery as an important differential diagnosis in patients with angina, ventricular arrhythmias, or even sudden cardiac death, especially in the younger population.

## Introduction

Coronary artery anomalies (CAA), though rare (0.01% to 0.03% incidence), carry a high risk of mortality and morbidity when present [1]. Anomalous origin of the left anterior descending artery (LAD) has been associated with myocardial infarction, heart failure, arrhythmias, cardiomyopathy, and complication during cardiac interventions and surgery [2]. Timely evaluation and intervention of the anomalies of LAD can help reduce the mortality and morbidity associated with it [3]. Here, we present a case of an incidental finding of anomalous LAD from the right coronary ostium in an 83-year-old female. Her chest discomfort was determined to be due to coexisting severe triple vessel disease. Thus, she underwent coronary bypass graft surgery with relief of symptoms.

## Case presentation

An 83-year-old female presented to the cardiac clinic with complaints of neck pain and upper back pain for the past few weeks, associated with exertion and difficulty walking on a treadmill. She had no associated chest pain or shortness of breath. Her initial physical examination was normal, with a blood pressure of 110/60mmHg and a heart rate of 60 beats/min. Her physical examination was unremarkable. Her past medical history included diabetes and hypertension, treated with antihypertensive and oral hypoglycemic agents. Her family history was significant for a daughter with myocardial infarction at 59 years and diabetes.

**Figure 1:**
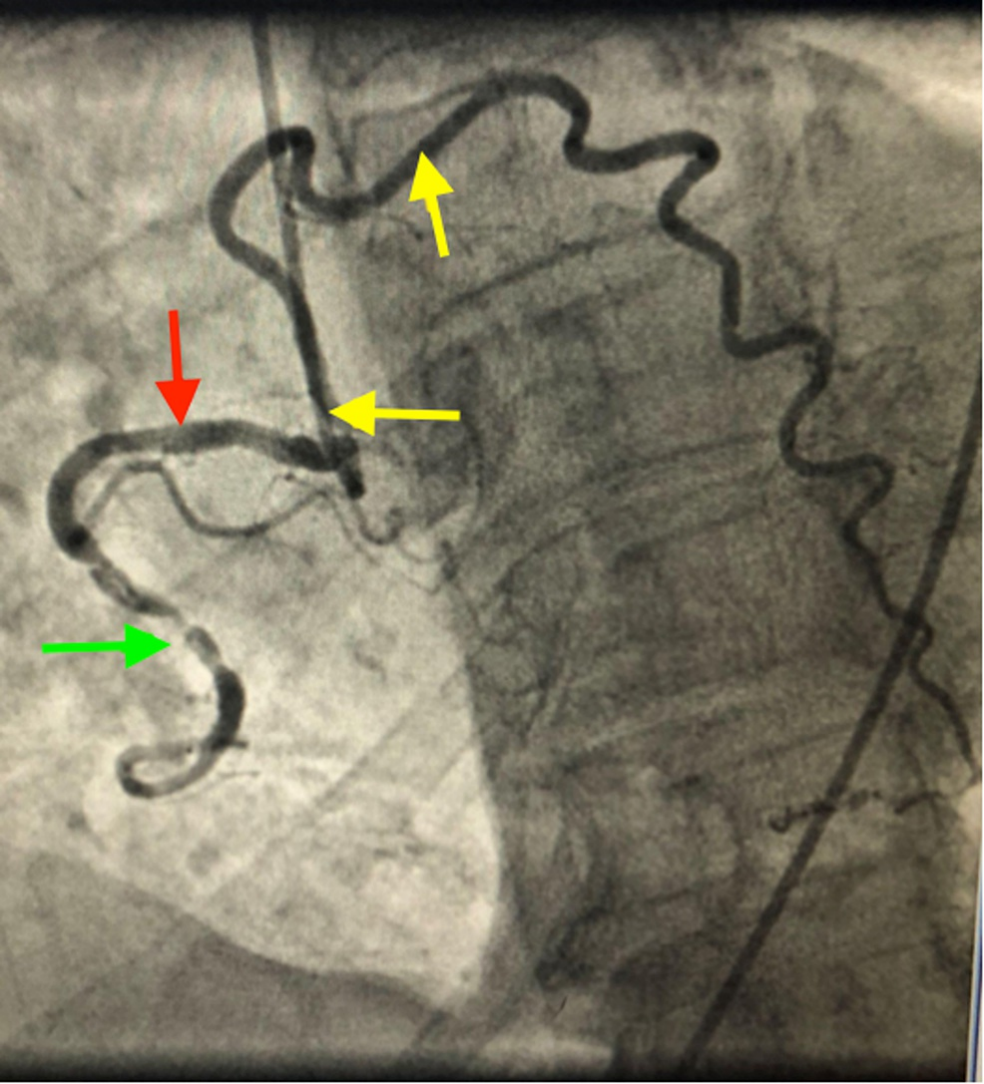
Coronary angiogram image showing type III (yellow arrow) left anterior descending artery arising from the right ostium (red arrow) supplying up to the apex. The right coronary artery shows multiple lesions (green arrow).

Her EKG showed normal sinus rhythm with no ST-T changes. Laboratory evaluation mild anemia with a hemoglobin of 9.7 g/dL (normal range: 12 to 15.5 g/dL), urea 52 mg/dL (normal range: 6 to 24 mg/dL) and normal creatinine levels. An echocardiogram showed no regional wall motion abnormality, mild concentric left ventricular hypertrophy, mildly dilated left atrium, mild to moderate mitral regurgitation, mild tricuspid regurgitation, and normal left ventricular systolic function with an ejection fraction of 55%-60%. The patient was provisionally diagnosed with Angina on exertion and essential primary hypertension. Since the patient could not walk on the treadmill, a pharmacological stress test with myocardial perfusion imaging was performed, which showed evidence of moderate to severe ischemia in the mid inferior region. Post-stress imaging, LV function was normal. Left heart catheterization was done. The origin of LAD was seen from the right ostium with 75% stenosis at the origin and then continued after traversing to the left side as a mid and distal LAD without any stenosis in mid and distal LAD. Right coronary arteriogram performed showed medium-sized codominant right coronary artery (RCA) multiple lesions (Figure 1).

**Figure 2:**
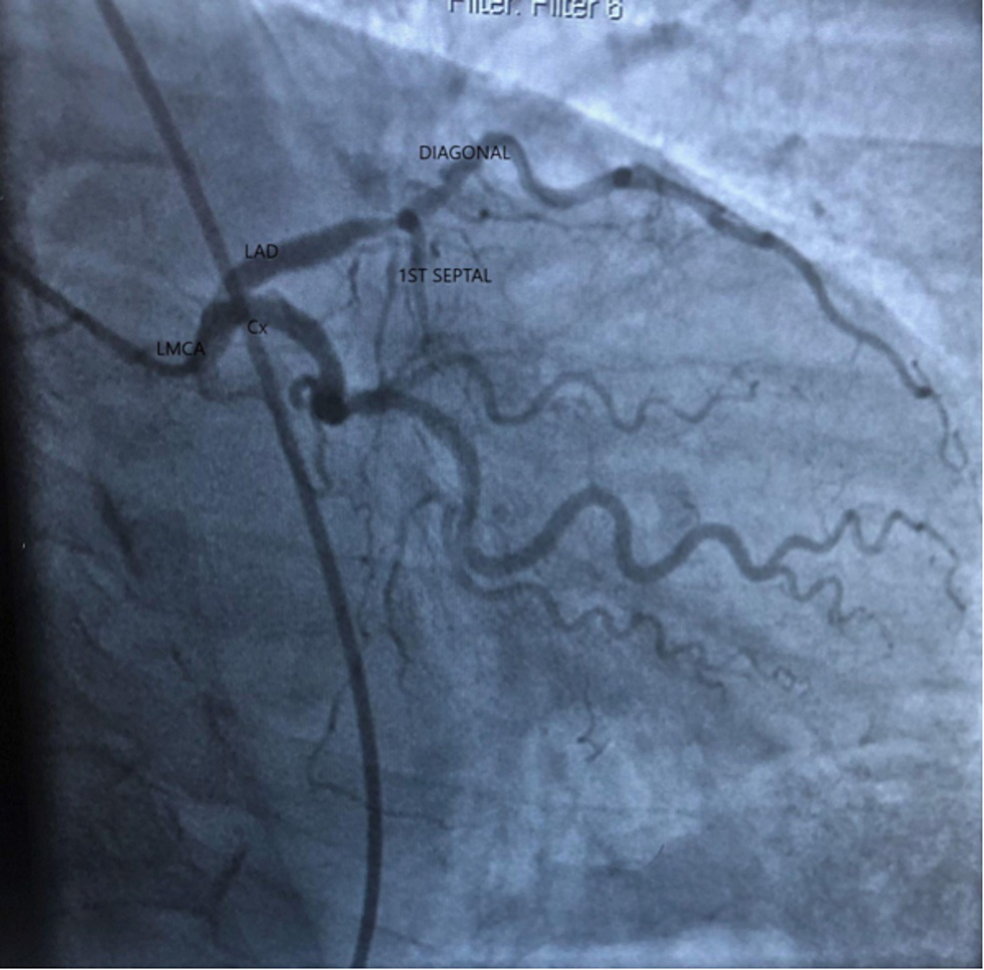
Angiographic image showing left main coronary artery (LMCA) dividing into circumflex (Cx) and left anterior descending artery (LAD). LAD is seen only up to the first part and then the diagonal branch is seen with 90% stenosis. Circumflex is seen with 80% to 90% stenosis.

Distal LAD was providing some collateral to the right coronary. The left main coronary artery (LMCA) is divided into LAD and circumflex (LCx). LAD was only seen up to the first septal, and then there was a diagonal branch with 90% stenosis (Figure 2).

The patient was diagnosed with severe triple vessel coronary artery disease with an abnormal origin of LAD from the RCA. The patient was referred to cardiac surgery for coronary artery bypass graft (CABG). After the thorough preoperative assessment, the patient underwent CABG with four grafts. The postoperative period was uneventful.

## Discussion

CAA are defined as morphological, anatomical variants found in <1% of the population [1]. They are classified based on origin, course, termination, and hemodynamic significance [4]. CAA anomalies can be benign or malignant. The LAD and LCx arising from the left sinus of Valsalva, LCx from the right sinus of Valsalva (RSV), the ectopic origin of coronaries from the posterior sinus of Valsalva (PSV), origin from ascending aorta, absent circumflex, intercoronary communication, and small coronary artery fistula is considered benign [2]. Malignant anomalies include either ectopic origin from the pulmonary artery (PA) or opposite aortic sinus, single coronary artery and large coronary artery fistulae [4]. The non-atherosclerotic mechanisms causing malignant risks include small artery, high acute take-off, compression of LAD, myocardial squeezing, and vasospasm. The studies by Angelini et al have determined that coronary hypoplasia, lateral compression, and stenotic segment length play role in the severity of interarterial anomaly with proximal intussusception of the aortic root [1].

The LAD normally originates as a bifurcation from LMCA continues diagonally towards the apex in the anterior interventricular sulcus giving diagonal and septal branches. Congenital abnormality of the origin or course of a coronary artery that arises from the aorta is known as the anomalous aortic origin of the coronary artery (AAOCA). Anomalous LAD can have its origin from RCA or RSV or PSV [5]. The anomaly of LAD originating from RCA is sometimes associated with congenital heart defects such as the tetralogy of Fallot. The uneven septation of CONUS and truncus arteriosus and hypoplasia of the pulmonary infundibulum is most often associated with the anomalous origin of LAD from the RCA in tetralogy of Fallot. LAD originating from the RCA can take various courses such as prepulmonic, interarterial, subpulmonic, retro aortic, and retro cardiac [5]. The interarterial or intramural course is more malignant than other anomalies [6]. The various clinical presentations of AAOCA include chest discomfort, angina, heart failure, arrhythmias, and sudden cardiac death. The risk of sudden cardiac death is increased in athletes and military recruits. Sudden cardiac death is more common among young patients. Heart failure, myocardial ischemia, and arrhythmias are common among the elderly [7], which could be attributed to aortic wall stiffness which increases with age [8]. In our case, anomalous LAD did not have an interarterial course that was contributing to her symptoms. We ruled this out by coronary angiogram. She had symptoms of angina on exertion due to atherosclerotic multivessel coronary artery disease. 

CAA can be diagnosed using echocardiography, coronary CT angiography, magnetic resonance angiography, and catheter angiography. Echocardiography [6] is very useful in children to detect coronary Ostia and the proximal courses of arteries in children, but limited to use in adults due to operator dependence and poor acoustic window leading to more false negatives. Catheter angiography was the standard investigation for detecting CAA before the advent of CT and MR imaging [9]. CT and MR imaging with or without stress/perfusion are non-invasive tests. The choice of each test depends on local resources and patient factors. Coronary angiography, using catheterization, CT, or MR, is recommended to evaluate anomalous coronary arteries [10]. Sudden onset syncope, chest pain, ventricular arrhythmias, and cardiac arrest especially in a younger patient should prompt a clinician to consider above-mentioned modalities to rule out anomalous coronary artery as an important differential diagnosis. In elderlies, it could also be found as an incidental finding as evident in our case.

Further, the use of intravascular ultrasound (IVUS) [9] can aid in measuring parameters such as coronary hypoplasia, lateral compression, and the severity of stenosis along the length of the anomalous vessel through different phases of the cardiac cycle. This would help in determining the need and type of feasible intervention [7]. The management of AAOCA depends on whether the patients are asymptomatic or symptomatic, ALAD or ARCA, ischemic changes on diagnostic testing, and the artery course. Symptomatic patients are recommended to undergo surgical intervention such as coronary artery bypass grafting and marsupialization of the coronary artery. Osteoplasty, reimplantation, and PA translocation are surgical techniques for AAOCA without an intramural course. In patients with ischemia, intracoronary stents can also be used as an alternative. Asymptomatic patients with ARCA/ALAD are conservatively managed with observation unless ischemic on diagnostic testing. Our patient here had a normal EKG, and the stress myocardial perfusion scan showed moderate to severe ischemia in the mid inferior region. Since the patient was symptomatic with angina on exertion and coronary angiography showed severe triple vessel disease with an ectopic origin of LAD from the RCA, the patient was advised to undergo CG with grafts. Asymptomatic patients at risk of SCD such as young athletes and military recruits can be provided with exercise guidelines, counseling for preparedness for cardiac events. Exercise restriction in all patients with AAOCA is not advised due to long-term detrimental effects. Based on the type of anomaly and subsequent risk stratification, patients can be provided with exercise guidelines [11].

## Conclusions

Anomalous origin of the left coronary artery from the right coronary ostium is an infrequently seen entity. Depending on its course after the origin, it may pose a significant risk of myocardial ischemia, ventricular arrhythmias, or sudden cardiac death. Younger patients might be more prone to such fatal consequences especially if the left coronary artery takes interarterial course between the aorta and PA and poses a risk of compression. In the elderly, calcification of the aorta might make anomalous left coronary artery more vulnerable to compression. Incidental findings of the anomalous left coronary artery without a dangerous course should prompt a clinician to look for an alternate cause such as atherosclerotic coronary artery disease as evident in our case.
